# Basolateral Sorting of the Sodium/Iodide Symporter Is Mediated by Adaptor Protein 1 Clathrin Adaptor Complexes

**DOI:** 10.1089/thy.2022.0163

**Published:** 2022-10-14

**Authors:** Petrina Koumarianou, Celia Fernández-Méndez, Dánae Fajardo-Delgado, Lidia Mirella Mielu, Pilar Santisteban, Antonio De la Vieja

**Affiliations:** ^1^ Instituto de Investigaciones Biomédicas “Alberto Sols”, Consejo Superior de Investigaciones Científicas (CSIC), Universidad Autónoma de Madrid, Madrid (UAM), Spain.; ^2^ Unidad de Tumores Endocrinos (UFIEC), Instituto de Salud Carlos III (ISCIII), Madrid, Spain.; ^3^ Ciber de Cáncer (CIBERONC), Instituto de Salud Carlos III (ISCIII), Madrid, Spain.

**Keywords:** AP-1A, AP-1B, clathrin, NIS trafficking, protein sorting, sodium iodide symporter

## Abstract

**Background:**

The sodium/iodide symporter (NIS) is a transmembrane protein located on the basolateral membrane of thyrocytes. Despite its physiological and clinical relevance, little is known about the mechanisms that mediate NIS subcellular sorting. In the present study, we examined NIS basolateral trafficking *in vitro* using non-thyroid and thyroid epithelial cells.

**Methods:**

Immunofluorescence and Western blotting were performed to analyze NIS subcellular location and function in cells grown in monolayers under unpolarized and/or polarized conditions. Strategic NIS residues were mutated, and binding of NIS to clathrin adaptor complexes was determined by immunoprecipitation.

**Results:**

We show that NIS reaches the plasma membrane (PM) through a thyrotropin-dependent mechanism 24 hours after treatment with the hormone. We demonstrate that NIS basolateral trafficking is a clathrin-mediated mechanism, in which the clathrin adaptor complexes AP-1 (A and B) sort NIS from the *trans*-Golgi network (TGN) and recycling endosomes (REs). Specifically, we show that the AP-1B μ1 subunit controls NIS basolateral sorting through common REs. In its absence, NIS is apically missorted but remains functional. Additionally, direct NIS basolateral transport from the TGN to the basolateral membrane is mediated by AP-1A through clathrin-coated vesicles that also carry the transferrin receptor. Loss of the μ1 subunit of AP-1A is functionally compensated by AP-1B. Furthermore, loss of both subunits diminishes NIS trafficking to the PM. Finally, we demonstrate that AP-1A binds to the L121 and LL562/563 residues on NIS, whereas AP-1B binds to L583.

**Conclusions:**

Our findings highlight the novel involvement of the clathrin-coated machinery in basolateral NIS trafficking. Given that AP-1A expression is reduced in tumors, and its expression correlates with that of NIS, these findings will help uncover new targets in thyroid cancer treatment.

## Introduction

The sodium/iodide symporter (NIS), encoded by the *SLC5A5* gene, mediates iodide uptake for the synthesis of thyroid hormones.^1–4^ NIS is typically localized to the basolateral membrane of polarized thyroid follicular cells; however, it can be expressed both at the basolateral membrane and at the apical membrane depending on its function in uptake or reabsorption, which are tissue-specific features.^4–9^ NIS is mostly localized intracellularly in thyroid tumors, preventing successful radioiodide (RAI) therapy.^7,9,10^ *SLC5A5* gene expression and NIS location in the membrane of thyroid follicular cells are positively regulated by thyrotropin (TSH) and other factors,^9,11–15^ whereas activation of the RET-RAS-BRAF pathway suppresses NIS expression and membrane localization by different signaling cascades.^16–18^

While the crucial role of NIS location for its activity is well demonstrated,^4^ the mechanism(s) underlying NIS trafficking to the basolateral and apical membrane remains enigmatic. A better understanding of the control of NIS expression and trafficking is important not only for thyroid gland physiology but also for recovering NIS function in cancer, which is critical for the success of ablative RAI. In this line, several proteins have been identified to interact with NIS and participate in thyroid cancer, including leukemia-associated RhoGEF (LARG),^19^ pituitary tumor transforming gene binding factor,^20,21^ ADP ribosylation factor 4 (ARF4),^22^ and valosin-containing protein.^22^

Basolateral sorting signals are simple peptide motifs in the cytoplasmic domain of proteins that are recognized by adaptor proteins. This includes tyrosine, monoleucine, dileucine residues, and PDZ-binding motifs.^23^ NIS is reported to have a PDZ target motif and three acidic dipeptide motifs in its C-terminus.^24^ Intriguingly, the cytoplasmic C-terminus of NIS contains a dileucine motif that can be recognized by clathrin adaptors,^24,25^ which might be involved in NIS trafficking.

Cytosolic adaptor proteins are components of protein coats associated with the *trans*-Golgi network (TGN) and/or recycling endosomes (REs), which may link cargo binding to clathrin.^26^ The clathrin-associated adaptor protein 1 (AP-1) complex has a major role in clathrin-mediated basolateral sorting. AP-1 is a heterotetrameric complex that is present in two homologous isoforms, AP-1A and AP-1B, which share three of four subunits (β1, γ, and σ) and differ only in the subunits μ1A and μ1B, respectively.^27^ Whereas AP-1A preferentially functions in biosynthetic routes, AP-1B sorts basolateral plasma membrane (PM) proteins in both biosynthetic and recycling routes.^28–31^ In this regard, a recent study reported that AP-1B recognizes the NIS C-terminus.^32^

In this study, we sought to investigate the potential NIS-trafficking role of the adaptor protein complex AP-1 and to evaluate whether a clathrin-mediated pathway is responsible for NIS basolateral trafficking.

## Materials and Methods

### Cell culture, transfection, and immunofluorescence

Non-polarized rat PCCl3 thyroid differentiated follicular cells and polarized Madin-Darby canine kidney (MDCK) cells were maintained as described.^14,33^ Gene silencing, transfection, and immunofluorescence protocols are described in the Supplementary Data.

### Plasmids and site-directed mutagenesis

Plasmids and primers used for site-directed mutagenesis are detailed in the Supplementary Data.

### Protein extraction and detection

All experimental techniques for protein extraction and detection are described in the Supplementary Data.

### RAI transport assay and co-immunoprecipitation

Polarized MDCK-hNIS and μ1AB-KD-hNIS cells were assayed for iodide transport as described,^34^ with modifications detailed in the Supplementary Data.

## Results

### NIS is trafficked from the TGN to the PM in clathrin-associated AP-1 vesicles and transferrin receptor-positive endosomes

**FIG. 1.**
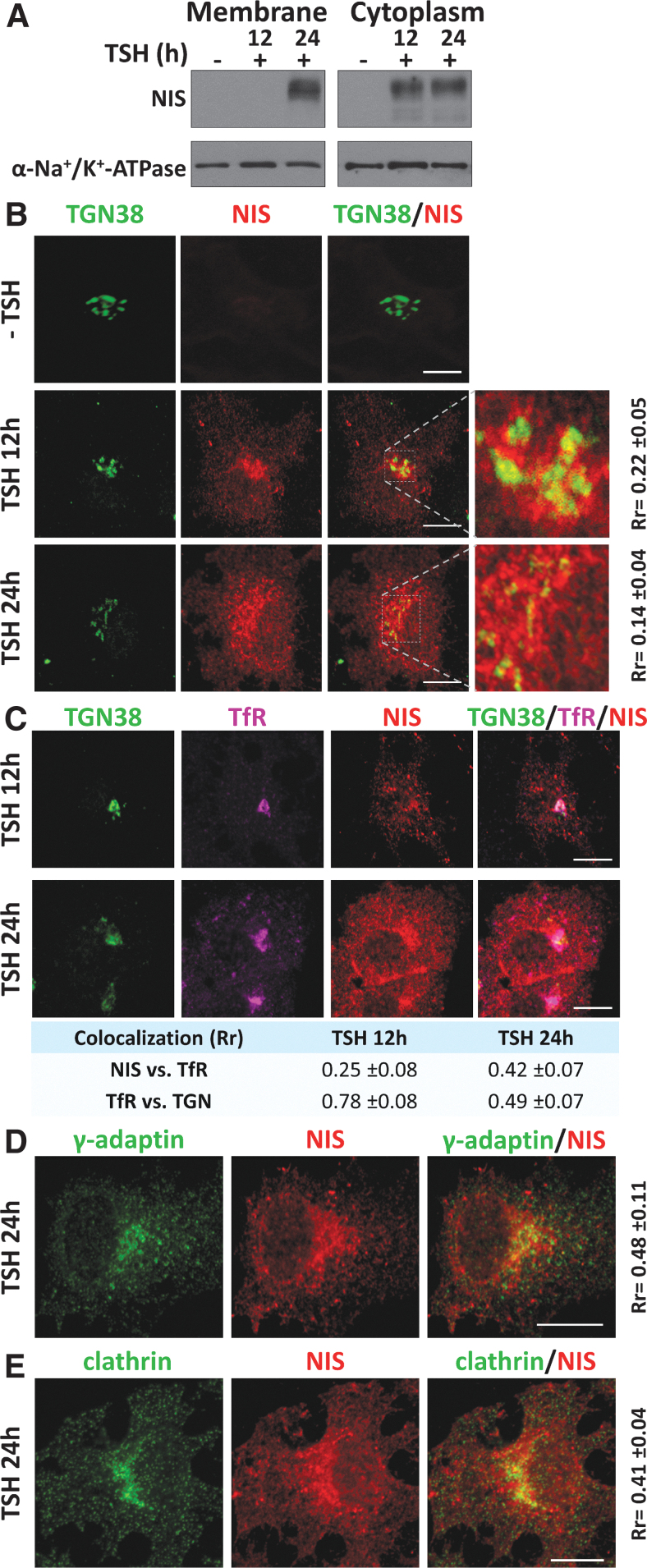
Cellular localization of newly synthesized NIS in PCCl3 cells after TSH induction and its localization within the TGN and recycling endosomes. (**A**) NIS protein expression by Western blotting of cytoplasmic and membrane-biotinylated extracts of PCCl3 cells grown in medium without TSH during 5 days (−) and then stimulated with TSH for 12 and 24 hours. α-Na^+^/K^+^-ATPase was used as a loading control. (**B**) Colocalization of NIS (red) with TGN38 (green) at the TGN in 5-day TSH-depleted PCCl3 cells (−TSH) and after repletion with TSH for 12 and 24 hours. White squares mark an expanded area shown in the inset (right panels). (**C**) Triple colocalization confocal microscopy of NIS (red), TGN38 (green), and recycling endosome TfR (magenta) markers in PCCl3 cells after 12 and 24 hours of TSH treatment. (**D, E**) Confocal immunofluorescence images of NIS colocalization with γ-adaptin (**D**) and clathrin (**E**) after 24 hours of TSH treatment. Scale bars, 10 μm. Quantitative colocalization analysis is indicated by Pearson's correlation coefficient (*R*_r_) and appears either in a blue table or in the lateral side of the colocalization images. More than 10 cells were analyzed in each case, and values are expressed in mean ± SD. NIS, sodium/iodide symporter; TfR, transferrin receptor; TGN, *trans*-Golgi network; TSH, thyrotropin.

To characterize the basolateral sorting routes for NIS, we first assessed the subcellular location of newly synthesized NIS in cultured PCCl3 cells upon TSH treatment. The absence of TSH led to the complete loss of NIS at the PM and cytoplasm (Fig. 1A), whereas its addition to the culture medium induced cytoplasmic NIS expression after 12 hours, which was detectable in the PM after 24 hours (Fig. 1A). Microscopy analysis revealed the presence of NIS in the TGN after 12 hours of TSH stimulation, as evidenced by its colocalization with the *trans*-Golgi protein TGN38 (Fig. 1B). NIS expression was also evident in the perinuclear region and cytoplasm, but not at the PM. NIS was confirmed at the PM after 24 hours of treatment, accompanied by a strong perinuclear and cytoplasmic distribution, and with little overlap with TGN38 (Fig. 1B). NIS labeling at a region close to the TGN suggested its association with REs, which transport cargo from the Golgi to the PM dependent on the clathrin-associated adaptor complex AP-1.^28,35^

NIS localization at REs was confirmed by analyzing the RE marker transferrin receptor (TfR), which is classically sorted by a clathrin-dependent mechanism. We observed an increase in NIS colocalization with TfR after 24 hours of TSH treatment, concomitant with a decline in colocalization between TfR and TGN38 (Fig. 1C).

Immunofluorescence analysis of NIS and the gamma subunit of AP-1 (γ-adaptin) (Fig. 1D), as well as with clathrin (Fig. 1E), revealed colocalization at TGN/RE regions 24 hours after TSH supplementation, and a few areas of colocalization were also observed in the cytoplasm.

Overall, these results suggest that NIS exits the TGN and follows a clathrin-dependent biosynthetic pathway to the PM in thyroid follicular cells mediated by AP-1-coated vesicles and TfR-rich REs. Because γ-adaptin is a common subunit of AP-1A and 1B complexes, and TfR interacts with both at the TGN and at REs,^31^ we explored the role of each clathrin adaptor variant in this process.

### NIS colocalizes with the γ, μ1A, and μ1B subunits of AP-1 at the TGN and the perinuclear region

**FIG. 2.**
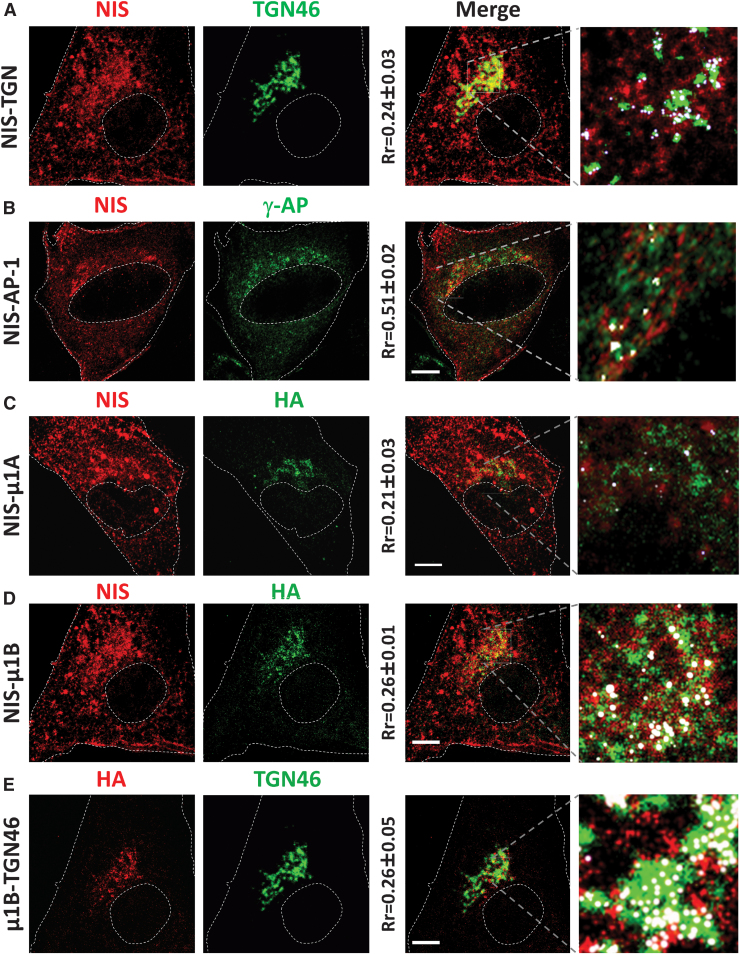
NIS colocalization with clathrin adaptors in MDCK cells. MDCK-hNIS cells were transfected with μ1A-HA or μ1B-HA vectors, and protein colocalization was analyzed by immunofluorescence after 48 hours. Shown are single representative confocal sections labeled with NIS (red), TGN46 (green), and HA (green/red). NIS colocalization with (**A**) TGN46, (**B**) γ-adaptin (γ-AP), (**C**) clathrin adaptor subunits μ1A (HA), and (**D**) μ1B (HA). (**E**) TGN46 colocalization with μ1B (HA). Scale bars, 10 μm. Regions of interest in white box are amplified, and colocalization points are shown as white dots in the inset (right panels). Quantitative colocalization analysis is indicated by Pearson's correlation coefficient (*R*_r_). More than 10 cells were analyzed in each case, and values are expressed in mean ± SD. Dashed lines have been added to indicate the location of the edges of the nucleus and the PM. HA, human influenza hemagglutinin tag; MDCK, Madin-Darby canine kidney; PM, plasma membrane.

To investigate where NIS basolateral sorting occurs, we performed immunofluorescence analysis for NIS and the TGN marker TGN46 in non-polarized MDCK cells overexpressing human NIS (MDCK-hNIS) (Fig. 2A). Quantitative colocalization analysis revealed a small overlap between NIS and TGN46, and NIS was also present at the perinuclear region and the PM. The intense NIS labeling at the perinuclear region, close to the TGN, again suggested its association with the REs that transport cargo from the Golgi to the PM dependent on AP-1 adaptors.^28,35^

We thus examined colocalization between NIS and the endogenous γ-adaptin (Fig. 2B) in MDCK-hNIS cells also overexpressing μ1A (Fig. 2C) or μ1B (Fig. 2D) proteins. Confocal microscopy results showed a weak colocalization between μ1A and NIS at the TGN (white dots in the inset in Fig. 2C). Qualitative and quantitative analysis revealed small differences in colocalization between NIS and TGN46 (Fig. 2A) and overexpressed μ1B (Fig. 2D). Partial colocalization was also observed between the μ1B-positive compartment and the TGN (Fig. 2E).

These data show that NIS colocalizes with AP-1A and AP-1B clathrin adaptor carriers at the TGN and RE, pointing to their involvement in NIS sorting.

### Knockdown of the AP-1A μ1A subunit has no effect on NIS basolateral membrane localization in polarized MDCK cells

**FIG. 3.**
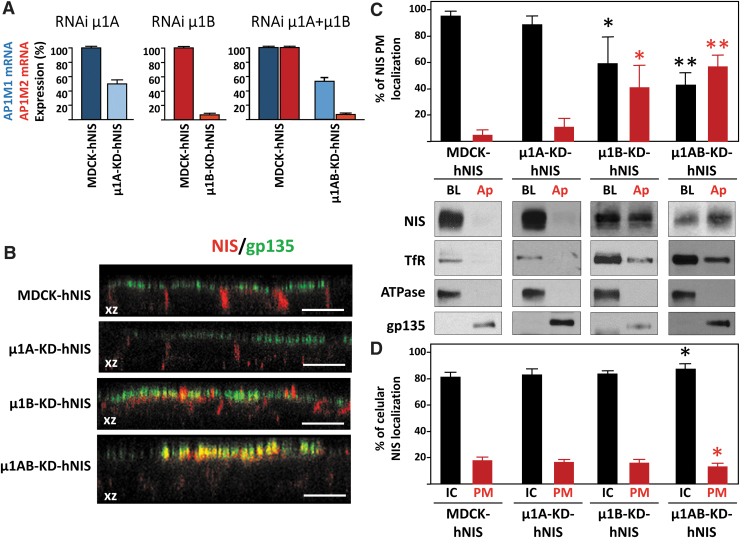
NIS localization in the absence of μ1A, μ1B, or both. (**A**) *AP1M1* and/or *AP1M2* mRNA levels were determined by RT-qPCR in control MDCK-hNIS cells and in μ1A RNAi knockdown (μ1A-KD-hNIS), μ1B knockdown (μ1B-KD-hNIS), and μ1A+B knockdown (μ1AB-KD-hNIS) cells. (**B**) MDCK-hNIS, μ1A-KD-hNIS, μ1B-KD-hNIS, and μ1AB-KD-hNIS cells were plated on polycarbonate filters and analyzed for the polarized distribution of NIS. Representative confocal immunofluorescence orthogonal *xz* plane views stained for NIS (red) and the apical glycoprotein gp135 (green) are shown. Scale bars, 10 μm. (**C**) Cells plated on polycarbonate filters were analyzed for the polarized distribution of NIS by biotinylation. Representative Western blots of NIS, TfR, α-Na^+^/K^+^-ATPase, and gp135 proteins in basolateral (BL) and apical (Ap) domains are shown. The upper part shows the quantification of the NIS signal in each domain as the percentage of the total PM protein (Ap+BL). (**D**) Quantification of NIS localization IC vs. PM (BL+Ap). In (**C, D**) data represent the mean ± SD for at least three experiments. Differences vs. control (MDCK-hNIS) were considered significant: *p < 0.05; **p < 0.01. IC, intracellular; RT-qPCR, quantitative reverse transcription polymerase chain reaction.

AP-1A controls trafficking from the TGN to the basolateral membrane by interactions between the subunit μ1A and cargo proteins through canonical and noncanonical motifs.^30,31^ We assessed the role of AP-1A in NIS sorting by silencing the expression of μ1A in polarized MDCK-hNIS cells (grown on filters) (Fig. 3A). No changes were observed for basolateral NIS localization in polarized μ1A-KD-hNIS (50% silenced) cells compared with the steady-state basolateral NIS localization in control MDCK-hNIS cells (Fig. 3B). These findings were confirmed by biotinylation and Western blotting (Fig. 3C), which revealed a similar amount of NIS in the basolateral domain of both control (95% ± 3%) and μ1A-KD-hNIS (89% ± 7%) polarized cells and practically no apical NIS distribution. Basolateral membrane α-Na^+^/K^+^-ATPase and apical membrane gp135 were analyzed to confirm polarization in μ1A-silenced cells (Fig. 3C).

These results suggest that AP-1A does not participate in NIS sorting, with the caveat that the degree of μ1A silencing was modest. In addition, it is known that AP-1B can compensate for the loss of available AP-1A.^31^

### Knockdown of the μ1B subunit of AP-1B missorts NIS at the apical membrane of polarized MDCK cells

We next examined the role of the epithelial-specific AP-1B variant, which interacts through its μ1B subunit with conventional tyrosine-based and unconventional signals localized on the cytoplasmic tail of cargo proteins.^30,31,36^ Confocal microscopy revealed an apical redistribution of NIS in μ1B-knockdown MDCK-hNIS cells (μ1B-KD-hNIS) that contrasted dramatically with its exclusive basolateral localization in control MDCK-hNIS cells (Fig. 3B). Nevertheless, the morphology of tight junctions (ZO-1) and adherens junctions (β-catenin) and polarization were preserved (Supplementary Fig. S1). Biotinylation analysis confirmed a decrease in the steady-state basolateral polarity of NIS in μ1B-KD-hNIS cells (61% ± 15%) and an increase in apical polarity (39% ± 15%), which was accompanied by a decrease in the basolateral distribution of TfR (Fig. 3C). Conversely, α-Na^+^/K^+^-ATPase and gp135 displayed normal polarity, in agreement with previous observations.^28^

Overall, these results show that AP-1B is likely involved in NIS sorting to the basolateral membrane.

### Double knockdown of μ1A and μ1B provokes severe NIS missorting and demonstrates the involvement of AP-1A in basolateral NIS trafficking

Our findings so far suggest that only AP-1B mediates NIS basolateral sorting. However, it is known that AP-1B can compensate for AP-1A function during basolateral sorting and membrane targeting of TfR and the low-density lipoprotein (LDL)-receptor.^31^ To clarify the role of AP-1A in NIS basolateral trafficking, we analyzed the polarized distribution of NIS in cells silenced for both μ1A and μ1B subunits (μ1AB-KD-hNIS) (Fig. 3). Confocal microscopy revealed strong apical NIS staining and colocalization with gp135 (Fig. 3B), and biotinylation assays demonstrated that 43% ± 5% of NIS was at the basolateral domain in μ1AB-KDhNIS cells, whereas 57% ± 5% was apically mistargeted (Fig. 3C). Conversely, TfR, α-Na^+^/K^+^-ATPase, and gp135 localization was unimpaired. The more conspicuous NIS delocalization in μ1AB-KD cells compared with μ1B-KD cells indicates that the AP-1A complex also participates in NIS basolateral sorting and mediates correct NIS polarized PM distribution through its μ1 subunit.

When we quantified the intracellular versus PM (basolateral+apical) localization of NIS (Fig. 3D), we found a small but significant increase in intracellular NIS (86.4% ± 2.1% vs. 81.5% ± 1.4%) in μ1AB-KD-hNIS cells compared with MDCK-hNIS cells (Fig. 3D), and a decrease of NIS in the PM (13.6% ± 2.1% vs. 18.5% ± 1.4%). This was also observed by immunofluorescence microscopy (Supplementary Fig. S2).

These results demonstrate that both AP-1A and AP-1B mediate NIS basolateral sorting at the TGN and at REs, and both are involved in NIS trafficking. Moreover, the decrease/absence of their expression reduces the amount of NIS reaching the PM.

### Mislocalized NIS at the apical membrane is functional

**FIG. 4.**
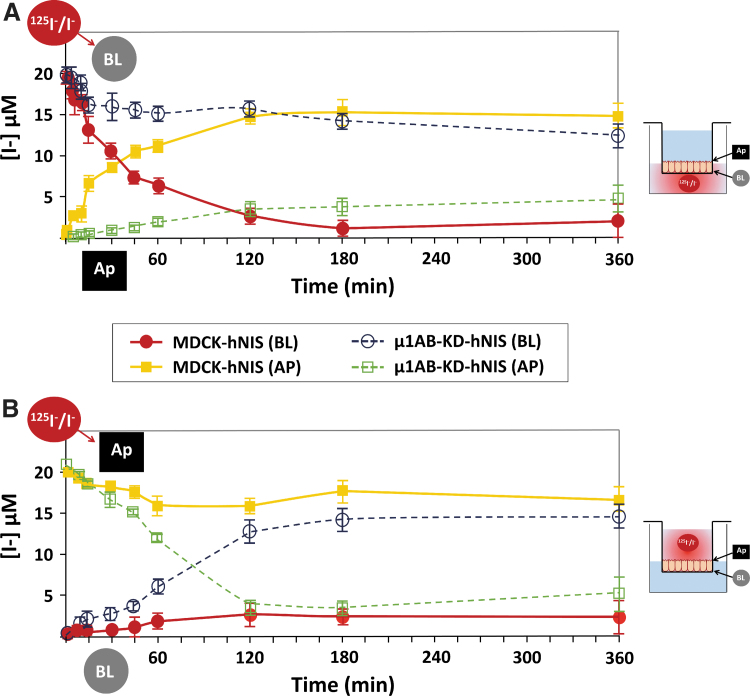
Apical-localized NIS is functional in μ1AB-KD-hNIS cells. Vectorial iodide transport in fully polarized MDCK-hNIS and μ1AB-KD-hNIS cells was measured in both chambers at the indicated time points. Iodide (20 μM) supplemented with ^125^I^−^ (100 mCi/mmol I) was added to the (**A**) basolateral (BL) or (**B**) apical (Ap) chamber, and 10 μL of medium was collected from each chamber at the time points shown. Radioactivity was quantified and iodide concentration was determined. Two different experiments with replicates were performed. BL (red line, solid circles) and Ap (yellow line, solid squares) iodide concentration in MDCK-hNIS cells. BL (dotted blue line, empty circles) and Ap (dotted green line, empty squares) iodide concentration in μ1AB-KD-hNIS cells.

Given that NIS mislocalization has been associated with impaired function,^34^ we next questioned whether the apically localized NIS remained functional and could transport iodide. We thus performed a timecourse iodide uptake assay in Transwell chambers using polarized MDCK-hNIS and μ1AB-KD-hNIS cells. Results showed that iodide added to the basolateral chamber was progressively transported to the apical chamber in MDCK-hNIS cells. By contrast, only a fraction of the iodide was transported from the basolateral to the apical chamber in μ1AB-KD-hNIS cells (Fig. 4A). We then evaluated the reverse reaction by adding iodide to the apical chamber.

Whereas little iodide was transported from the apical to the basolateral chamber in MDCK-hNIS cells, the bulk of iodide was transported from the apical to the basolateral chamber in μ1AB-KD-hNIS cells (Fig. 4B). The differences in the curve shape over time and in the amount of iodide transported by μ1AB-KD-hNIS (apical to basolateral) versus MDCK-hNIS (basolateral to apical) are presumably due to the occurrence of NIS on both sides of μ1AB-KD-hNIS cells. Overall, these results clearly demonstrate that the apically localized NIS is fully functional.

### Analysis of NIS interactions with clathrin adaptor (AP-1A and AP-1B)

**FIG. 5.**
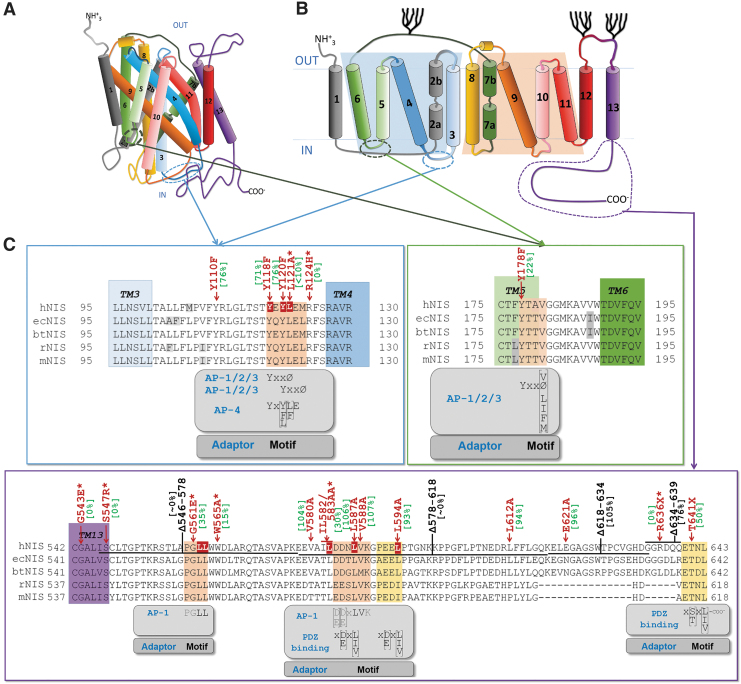
*In silico* localization of the clathrin adaptor binding sites in the cytoplasmic region of NIS protein, and functional implications. (**A**) Proposed 3D topology of NIS based on the crystal structure of *Vibrio parahaemolyticus* sodium/galactose symporter crystal structure.^40^ (**B**) NIS secondary structure model based on experimental findings.^38,39^ (**C**) Putative binding sites localize to the intracellular region and within the intracellular C-terminal region. We detected three tyrosine-based putative motifs in the intracellular loops. Motifs fitted the minimal consensus for YxxØ signals (where Ø is a bulky hydrophobic residue and X any amino acid) that are known to bind to the μ1A/μ1B subunits of AP-1A and AP-1B complexes, respectively.^30^ Two were localized in the intracellular region between the NIS TD 3 and 4: Y118[QE]YL121 and Y120LE[LM]123. The third was found at Y178T[AT]V181, between TD 5 and 6; however, these residues seem to be part of the TD6 helix according to the predicted model. Putative basolateral motifs that can be recognized by AP-1 were found in the C-terminal region: (i) a conserved dileucine motif PGLL (L562–L563), not previously reported^25,32^, and (ii) an LVK leucine-based motif preceded by a region of acidic residues, similar to the ALVVHP motif of syntaxin 4 that is responsible for its AP-1B-dependent basolateral localization.^57^ The amino acid binding sites are indicated by an orange square in NIS. The corresponding motif and the possible adaptors are indicated below the sequence. Residues of NIS that have been mutated and functionally analyzed are indicated over the amino acid sequence (red), together with the percentage of activity (green square).^3,25,32,34,41,54^ C-terminal deletions are also indicated (black). Additionally, an asterisk denotes that the indicated mutation has been determined to cause partial or total NIS mislocalization. AP-1, adaptor protein 1; TD, transmembrane domains.

We next sought to determine the interaction between AP-1 subunits and NIS. We first performed *in silico* and manual searching for possible binding sites^29,37^ using the proposed NIS topology (Fig. 5A, B).^38–40^ We identified three sites within the intracellular loops and two sites at the C-terminus. Additionally, we inspected the functional involvement of the surrounding region of those residues (Fig. 5C). Residues L121 and R124 in the loop between transmembrane domain 3 and 4 and C-terminal residues G561E, W565, and IL582/583AA disrupt NIS function,^25,41^ making them good candidates for interaction. Indeed, a previous computational simulation analysis predicted an interaction between NIS residue L583 and AP-1B.^32^

**FIG. 6.**
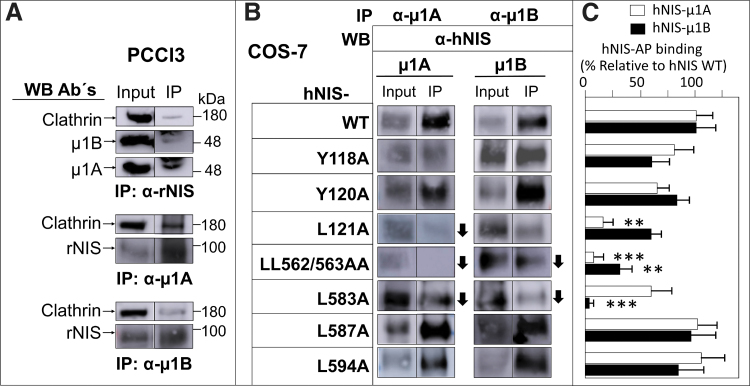
Analysis of NIS interaction with clathrin-dependent μ1A and μ1B. (**A**) Western blotting using the indicated antibodies (left) against PCCl3 input or IP complexes with rNIS (top), μ1A (middle), or μ1B (bottom) antibodies. (**B**) Western blotting of COS-7 cells cotransfected with WT hNIS or the indicated mutants (left) and with μ1A or μ1B, as indicated. Following IP with μ1A or μ1B, extracts were immunoblotted with a rabbit polyclonal anti-hNIS antibody. Input or IP fractions are indicated. The arrows highlight a decrease in the signal when compared with WT hNIS. The bands shown for each IP and Western blot are from the same membrane but were separated in the original Western blot. Western blots are representative of at least two experiments. (**C**) Estimation of the interaction of μ1A or μ1B with hNIS. The NIS signal obtained in the IP fraction was standardized to the input and then normalized to the interaction observed in the hNIS WT protein. Data represent the mean ± SD of at least three experiments. Differences vs. control (MDCK-hNIS WT) were considered significant: **p < 0.01; ***p < 0.001. IP, immunoprecipitated; WT, wild type.

We found an interaction between endogenous NIS and AP-1A/B subunits in PCCl3 cells by co-immunoprecipitation (Fig. 6A). Importantly, clathrin was also part of this protein complex.

We cotransfected COS-7 cells with wild-type hNIS and with mutations at residues Y118A, Y120A, L121A, LL562/563AA, L583A, L587A and L594A, plus μ1A (AP1M1) or μ1B (AP1M2) cDNAs to determine the specific binding sites of this interaction. Immunoprecipitation analysis with antibodies to μ1A/B revealed the presence of hNIS in almost all cases; however, the amount of NIS observed in the immunoprecipitated complexes with the point mutations differed according to the mutant analyzed (Fig. 6B). Specifically, the presence of hNIS protein in the μ1A immunoprecipitate was less in the case of the L121A and L583A mutants, and almost undetectable in the LL562/563A double mutant (Fig. 6C). In the μ1B immunoprecipitates, the interaction was weaker in the LL562/563A double mutant and in the L583A mutant, although to a lesser extent (Fig. 6C). These findings indicate that the aforementioned mutations do not completely block the interaction between NIS and AP-1A/B subunits, suggesting that binding relies on more than one site.

Taken together, our results demonstrate that AP-1A/B interacts with NIS in both endogenous and exogenous systems, polarized or not, and highlight the likely importance of the hNIS residues L121, LL562/563, and L583 for this interaction.

## Discussion

NIS is essential in thyroid physiology and forms the basis of RAI therapy.^3,4,42^ This therapy is, however, ineffective in some patients due to loss of NIS expression at both the level of mRNA^43,44^ and protein, and to intracellular retention,^7,10,45^ which may be caused by pertubations in NIS trafficking. When this occurs, patients are classified as RAI refractory. Nevertheless, given the success of RAI therapy in well-differentiated thyroid tumors, numerous targeted therapy strategies use NIS as a theragnostic tool.^3,4,46,47^ While the biological function of NIS in thyroid and non-thyroid tissues is well characterized, the mechanisms controlling its sorting and subcellular distribution have not been sufficiently explored. Accordingly, a better understanding of these processes should facilitate improvements in RAI-based therapies.

Basolateral sorting of integral membrane proteins is directed primarily by signals located in the cytoplasmic domain of the sorted protein.^22^ The heterotetrameric clathrin adaptor AP-1 has been implicated in basolateral sorting.^48,49^ AP-1A/B can function either at the TGN or in REs depending on where they encounter their preferred cargo. Using confocal microscopy, we show that AP-1A/B demonstrates comparable localization with NIS in PCCl3 cells. Indeed, they colocalize to similar extents with TGN/RE markers within the NIS trafficking route.

Loss of function, biotinylation, and confocal microscopy analysis reveal that AP-1A/B participate in NIS basolateral trafficking, although the role of AP-1A can be compensated by AP-1B. This phenomenon has been observed for basolateral sorting of the TfR and LDL receptors.^31^ The absence of both AP-1A/B subunits led to mislocalized basolateral NIS in MDCK cells, with a dominant apical expression, and also to a decrease in total NIS localized to the PM.

Using bioinformatics and manual searching, we found five putative binding sites for clathrin adaptor proteins (Fig. 5). Importantly, mutations in residues in the loop between transmembrane domains 3–4 have been previously implicated in NIS function and localization.^41^

The finding that NIS trafficking is clathrin-mediated might explain its different tissue-related location. The μ1A subunit is expressed in all cells,^50–52^ whereas the μ1B isoform is expressed differentially in some mammalian epithelial cells (e.g., distal kidney) where NIS localizes basolaterally,^4,7^ but not in other epithelial cells (e.g., kidney proximal cells) where NIS localizes apically.^4,6^

We also show that apically expressed NIS is functional following the elimination of the μ1AB isoform and can participate in iodide uptake from the apical space. Accordingly, NIS located apically in the proximal tube could participate in iodide reabsorption, as has been predicted.^4^

Site-directed mutagenesis and co-immunoprecipitation allowed us to identify the specific NIS residues that interact with μ1A (L121 and LL562/563) and μ1B (L583 and LL562/563). In most cases, the amino acid substitution did not completely abolish the interaction between NIS and AP-1. This is common to this type of approach^53^ and can be explained by the fact that the mutation only partially reduces binding affinity, or it might suggest the presence of multiple interaction sites. A previous study described that the C-terminus of NIS is involved in its trafficking (Fig. 5). A tryptophan acidic motif around W565 was suggested to interact with kinesin-1 light chain-2,^54^ in a region close to LL562/563, which are critical residues for μ1A binding as shown here. Also, by a modeling approach, they tentatively identified an interaction between μ1B and the L583 residue.^32^ Here, we confirm this interaction experimentally and provide a detailed map of the NIS residues involved in clathrin adapter binding.

**FIG. 7.**
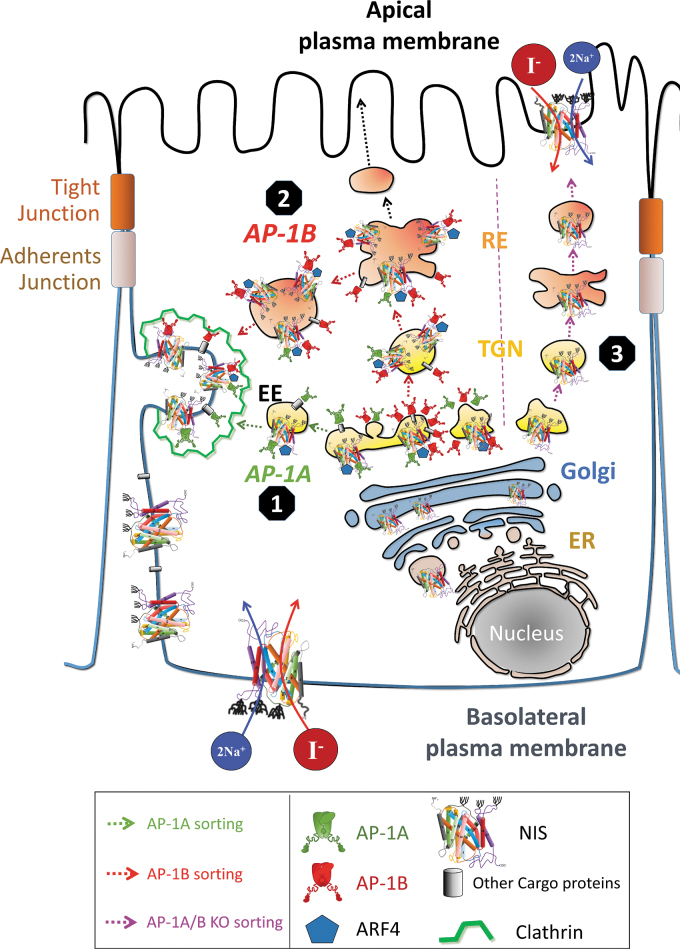
Model of NIS trafficking in epithelial cells. The AP complexes are widely distributed throughout the post-Golgi network. ❶ Ubiquitously expressed AP-1A is localized at the TGN and endosomes and regulates TGN-endosomal sorting and may directly regulate NIS TGN-basolateral PM sorting (AP-1A sorting, indicated in green lines). ❷ Epithelium-specific AP-1B is localized at the TGN and/or at the recycling endosomes and is crucial for the control of polarized NIS sorting to the basolateral PM (AP-1B sorting, indicated in red lines). ARF4 may be part of the clathrin-coated vesicles. ❸ Accordingly, the cargo (NIS) can be sorted to the lysosomal compartment or recycling. In the absence of AP-1A/B, NIS is delivered to the apical membrane (AP-1A/B KO sorting, indicated in purple lines). ARF4, ADP ribosylation factor 4; EE, early endosome; ER, endoplasmic reticulum; RE, recycling endosome.

Our characterization of NIS sorting and trafficking allows us to propose the following mechanism. After NIS synthesis in REs and total glycosylation in the Golgi apparatus, in polarized cells AP-1 adaptors bind NIS in the TGN, where AP-1A-enriched vesicles sort NIS directly to the PM. In addition, AP-1B-enriched vesicles translocate to REs and sort NIS to the PM in a clathrin-dependent mechanism (Fig. 7). In non-polarized cells, AP-1A/B contributes to NIS PM sorting.

These findings advance our understanding of NIS trafficking not only in epithelial cells in physiological conditions but also in non-polarized cells of refractory thyroid carcinomas where NIS fails to reach the PM and, hence, loses the ability to concentrate RAI. It will be important to analyze whether AP-1A/B proteins are altered in thyroid cancer. As a prelude to this analysis, we used the GEPIA webserver^55^ to perform a differential gene analysis of NIS (*SLC5A5*), μ1A (*AP1M1*), and μ1B (*AP1M2*) in samples of the Thyroid Cancer Genome Atlas.^56^ We found that *SLC5A5* and *AP1M1* are downregulated in tumor samples, and *AP1M2* is upregulated with respect to control tissue (Supplementary Fig. S3). Additionally, we found a positive correlation between *SLC5A* and both *AP1M1* and *ARF4*, another clathrin-dependent factor involved in NIS trafficking.^23^ Therefore, AP-1A/B might be a new target to increase NIS trafficking to the PM and improve RAI therapy in patients with refractory thyroid cancer.

## Supplementary Material

Supplemental data

Supplemental data

Supplemental data

Supplemental data
