# Supplementary material for: Basolateral Sorting of the Sodium/Iodide Symporter Is Mediated by Adaptor Protein 1 Clathrin Adaptor Complexes
Source: Thyroid. 2022 Oct 14;32(10):1259–70. doi: 10.1089/thy.2022.0163 (PMC9618391; doi:10.1089/thy.2022.0163)
Supplement: Supplemental data [file Supp_DataS1.docx]

**Supplementary Materials and Methods**

*Cell culture*

Non-polarized rat PCCl3 thyroid differentiated follicular cells were cultured and treated with TSH as described (1). Confluent cells were starved for TSH and insulin in the presence of 0.2% donor calf serum (starvation medium, indicated as minus [-] in the figures) for 5 days to completely abolish the expression of NIS, which has a half-life of ~3 days in the absence of TSH (2). TSH was added to the culture medium at 1 nM.

Polarized MDCK cells (strain II) were maintained as described (3); MDCK-hNIS cells stably expressed human NIS cDNA (4). The stable μ1B-KD MDCK cell line was stably silenced for the μ subunit of AP-1B protein (*AP1M2* gene) as described (5). The cells were then stably transfected with the pcDNA3.1-hNIS expression vector to generate the μ1B-KD-hNIS cell line. Clones resistant to geneticin (G418) were isolated and NIS expression levels were detected by western blotting. Three positive clones with different NIS expression levels were further analyzed in Transwell cultures by a membrane immunofluorescence assay. The results presented herein are all from the same clone. The clone was selected based on NIS expression levels given that NIS subcellular distibution was similar in all clones.

The MDCK-hNIS and μ1B-KD-hNIS cell lines were stably silenced for the μ subunit of AP-1A protein (*AP1M1* gene) using a lentiviral short hairpin RNA (shRNA) for *AP1M1* (pGIPZ, RHS4430-200279346); a nontargeting control (pGIPZ-shRNAmir-NS, RHS4346) was also constructed (both from Open Biosystems, Dharmacon, Lafayette, CO). Production of VSV-G pseudotyped lentivirus was performed as described (6). Cells were grown as described (3), supplemented with 0.6 mg/ml G418 (MDCK-hNIS cells) or together with 2.5 μg/ml puromycin (μ1B-KD-hNIS, μ1A-KD-hNIS and μ1AB-KD-hNIS cells). All cells were grown at 37°C and 5% CO_2_.

*Plasmids and site-directed mutagenesis*

The plasmids pBC6-mouse μ1A-HA (hemagglutinin) and pBC6-human μ1B-HA were provided by Dr. Heike Folsch (7). pcDNA3.1(-)-hNIS was provided by Dr. Nancy Carrasco (8). Site-directed mutagenesis was performed to create point mutations in the pcDNA3.1-hNIS expression vector using PfuUltra II Fusion HotStart DNA Polymerase (Agilent Technologies Inc., Santa Clara, CA) and complementary primers for the substitution (Supplementary Table 1). Mutagenesis was confirmed by sequencing.

**Supplementary Table 1.** Primers used for site-directed mutagenesis of the pcDNA3.1-hNIS expression vector.

| **Substitution (s)** | **Orientation** | **Sequence 5' - 3'** |
| --- | --- | --- |
|  |  |  |
| **Y118A** | Forward | TCACCAGCACCGCCGAGTACCTGGAGATG |
|  | Reverse | CATCTCCAGGTACTCGGCGGTGCTGGTGA |
| **Y120A** | Forward | AGCACCTACGAGGCACTGGAGATGC |
|  | Reverse | GCATCTCCAGTGCCTCGTAGGTGCT |
| **L121A** | Forward | CAGCACCTACGAGTACGCGGAGATGCGCTTCAGC |
|  | Reverse | GCTGAAGCGCATCTCCGCGTACTCGTAGGTGCTG |
| **LL562/563AA** | Forward | ACCCTGGCCCCGGGAGCGGCGTGGTGGGACCTCGC |
|  | Reverse | GCGAGGTCCCACCACGCCGCTCCCGGGGCCAGGGT |
| **L583A** | Forward | CAAGGAAGAAGTGGCCATCGCGGATGACAACTTGGT  CAAG |
|  | Reverse | CTTGACCAAGTTGTCATCCGCGATGGCCACTTCTTCCTTG |
| **L587A** | Forward | CCATCCTGGATGACAACGCGGTCAAGGGTCCTGAAG |
|  | Reverse | CTTCAGGACCCTTGACCGCGTTGTCATCCAGGATGG |
| **L594A** | Forward | ACTTGGTCAAGGGTCCTGAAGAAGCCCCCACTGGAAA |
|  | Reverse | TTTCCAGTGGGGGCTTCTTCAGGACCCTTGACCAAGT |

*Cell transfection*

Non-polarized kidney COS-7 (CRL-1651) cells were cultured according to the ATCC (https://www.atcc.org/products/crl-1651) and transfected using Lipofectamine 3000 (Invitrogen, Carlsbad, CA). For co-transfection, 8 µg of wild-type (WT) pcDNA3.1-hNIS or the mutants was co-transfected with 8 µg of pBC6-μ1A-HA or pBC6-μ1B-HA in a 10-cm plate. Transfected cells were assayed after 2 days.

*RT-PCR*

Total RNA was obtained using TRIzol Reagent (Thermo Fisher Scientific Inc., Waltham, MA). RNA was quantified and its purity was determined by measuring its absorbance at 260 nm and 280 nm using the Nanodrop ND1000 spectrophotometer (Thermo Fisher Scientific). One microgram of total RNA was retrotranscribed with the M-MLV Reverse Transcriptase (Promega Inc., Madison, WI) and RNase inhibitor (RNaseOUT, Invitrogen).

Silencing of *AP1M1* and *AP1M2* was confirmed by quantitative PCR (qPCR) performed in triplicate on the Mx3000P QPCR platform (Agilent Technologies). The qPCR followed a general protocol consisting of polymerase activation at 95˚C for 5 minutes followed by 40 cycles of denaturalization at 95˚C for 20 seconds, annealing at 60˚C for 20 seconds and extension at 72˚C for 30 seconds. All primers used were purchased from Sigma-Aldrich Chemical Co. (St. Louis, MO) and are listed in Supplementary Table 2. Primer specificity was determined by dissociation curves. Relative gene expression was calculated by the 2^−ΔΔCt^ method (9). Presented data are normalized to *GAPDH* expression.

**Supplementary Table 2.** Primers used for qRT-PCR.

| **Gene** | **Species** | **Orientation** | **Sequence 5' - 3'** |
| --- | --- | --- | --- |
| ***AP1M1*** | Dog | Forward | CCCTCAGCCACATCAGTGTT |
|  |  | Reverse | AAACATAGACAGGGTGGCGG |
| ***AP1M2*** | Dog | Forward | AGGTGTTTCTGTCGGGAATG |
|  |  | Reverse | AATTTCACGTCCTCCAGCTC |
| ***GAPDH*** | Dog | Forward | CATCAACGGGAAGTCCATCT |
|  |  | Reverse | ACTCAGCACCAGCATCACC |

*Immunofluorescence assays*

To monitor expression and/or the subcellular protein localization, cells were grown as monolayers on Transwell filters. Immunofluorescence analysis of monolayer unpolarized cells was performed as described (3) using the primary antibodies listed in Supplementary Table 3. Nuclei were stained with 4-,6-diamidino-2-phenylindole (DAPI) for 10 minutes and then coverslips were washed, dried, and mounted with Mowiol or Prolong Gold antifade reagent (Invitrogen). Single confocal sections of cells were examined with a laser-scanning TCS SP5 Leica confocal microscope (Leica Microsystems, Wetzlar, Germany), using a HCX PL APO lambda blue 63×/1.40 oil-immersion objective, 1024×1024 pixel resolution, and were processed with LAS AF Lite software (Leica). Further image processing was carried out with ImageJ (NIH). Quantitative colocalization analysis was performed with the ICA plugin (10) and the JACoP plugin (11) and expressed as Pearson’s correlation coefficient (Rr) and Manders’ coefficient (R). As both plugins gave the same Rr results, we show data derived only from the ICA plugin. More than 10 cells were analyzed for each condition and values are the mean±SD.

**Supplementary Table 3.** Antibodies.

| **Primary Antibody** | **Company** | **Nº Catalog** | **Immunogen** | **WB**  **dilution** | **IF**  **dilution** | **IP concentration** |
| --- | --- | --- | --- | --- | --- | --- |
| ɣ-adaptin | BD biosciences | 610385 | Mouse |  | 1:500 |  |
| AP1M1 | Sigma | SAB1301057 | Rabbit | 1:500 |  | 0.5 mg/ml |
| AP1M2 | Sigma | SAB2105800 | Rabbit | 1:500 |  | 0.5 mg/ml |
| β-catenin (C-18) | Santa Cruz | 1496 | Rabbit |  | 1:500 |  |
| Clathrin | BD biosciences | 610500 | Mouse |  | 1:500 |  |
| gp135 | From Dr. G. Ojakian | | Mouse | 1:50 | 1:200 |  |
| HA (16B12) | Covance | MM3-101P | Mouse |  | 1:100 |  |
| HA-FITC (3F10) | Roche | 11988506001 | Rat |  | 1:200 |  |
| α-Na+/K+-ATPase | Thermo | MA3-928 | Mouse | 1:500 |  |  |
| hNIS | From Dr. Nancy Carrasco | | Rabbit | 1:1000 |  |  |
| hNIS 21.3.4 | From Dr. Antonio De la Vieja | | Mouse | 1:200 | 1:200 | 0.5 mg/ml |
| hNIS (FP5A) | LabVision | FP5A | Mouse |  | 1:200 |  |
| rNIS | From Dr. Nancy Carrasco | | Rabbit | 1:1000 | 1:1000 |  |
| TfR | Invitrogen | 13-6890 | Mouse | 1:500 | 1:400 |  |
| TGN38 | AbD Serotec | AHP499G | Sheep |  | 1:800 |  |
| TGN46 | Merck | ABT95 | Rabbit |  | 1:200 |  |
| ZO-1 | Santa Cruz | 33725 | Rat |  | 1:200 |  |

Polarized cells grown on 12-mm Transwell filters were prepared for immunofluorescence analysis of membrane proteins according to the following protocol. Cells were fixed with 4% paraformaldehyde in phosphate buffered saline (PBS) containing 100 mM CaCl_2_ and 1 mM MgCl_2_ (PBSCM) for 30 minutes and the excess PFA was rinsed off with PBS. To gain access to intracellular epitopes, cells were permeabilized with 0.1% saponin in PBS at 4˚C for 15 minutes with gentle shaking, followed by two quick washes with PBS at room temperature (RT). Blocking was performed with 3% bovine serum albumin in PBS at RT for 30 minutes. The polycarbonate membranes were then excised from the Transwell inserts and incubated with specific primary antibodies (Supplementary Table 3) against the luminal domains of the respective antigens, overnight at 4˚C at pre-optimized dilutions in blocking solution. After washing off excess antibody, bound antibodies were detected with the appropriate combination of secondary antibodies at RT for 1 hour. Samples were mounted on microscope slides with Prolong Gold antifade reagent after nuclei staining (DAPI) for 5 minutes. All incubations and washes were performed on both sides of the insert. For the orthogonal views, a series of z-stacks (step 0.14 µm) were collected using a TCS SP5 Leica confocal microscope, as described for the immunofluorescence analysis.

*Protein detection methods*

Whole cell extracts were prepared as described (12). Levels of cell surface proteins were measured using a biotinylation assay (8) with the sulfo-NHS-SS-Biotin reagent (Pierce, Rockford, IL). Membrane, cytosolic and total extracts were collected during the process. Domain-selective biotinylation from the apical and basolateral domains was performed with polarized cells grown on 24-mm Transwell inserts. Cells were placed on ice and rinsed twice with ice-cold PBSCM (PBS containing 0.1 mM CaCl_2_ and 1 mM MgCl_2_) in both upper and lower compartments, and once with biotinylation buffer. Next, cells were incubated twice with 1.5 mg/ml sulfo-NHS-SS-Biotin in biotinylation buffer added either to the apical (0.9 ml) or to the basolateral (0.6 ml) side (while the opposite compartment was incubated with PBSCM) at 4˚C for 20 minutes. Cells were gently rinsed twice with biotinylation buffer and unreacted biotin was quenched using 100 mM glycine in PBSCM three times for 5 minutes. Filters were removed from the inserts and cells were lysed with 1 ml of Transwell lysis buffer composed of 50 mM Tris-HCl (pH 7.4), 150 mM NaCl, 25 mM KCl, 2 mM EDTA, 1% Na-deoxycholate, 0.1% SDS, 1% Triton X-100 and supplemented with protease inhibitors, for 30 minutes at 4˚C. Lysates were cleared and 0.2 ml was separated as the total protein fraction. Biotinylated proteins were retrieved from cleared lysates as described (8).

All samples were diluted in loading buffer and heated at 37˚C for 30 min. Samples were then separated by SDS-PAGE (1) and transferred onto nitrocellulose membrane (Schleicher & Schuell, Dassel, Germany), and immunoreactive proteins were visualized with the Pierce ECL western blotting substrate (Thermo Scientific). The primary antibodies used are listed in Supplementary Table 3. Protein expression levels were quantified using ImageQuant software (Molecular Dynamics, Sunnyvale, CA). The protein of interest was quantified and normalized in all cases to its loading control. Shown are representative blots from at least 3 independent experiments.

*Immunoprecipitation*

Primary antibody (2 µg) was incubated for 30 minutes at RT with 50 µl of pre-washed magnetic Dynabeads–Protein G (Thermo Scientific). Tubes were placed on a magnet and the supernatant was removed. Lysed total extract (0.2 mg) of PCCl3 or transfected COS-7 cells with WT hNIS or mutants and µ1A or µ1B in 500 µl of non-denaturing buffer (20 mM Tris-HCl pH 8.0, 137 mM NaCl, 1% Nonidet P-40 and 2 mM EDTA) were added to 100 µl of the Dynabeads-Antibody complex, and gently mixed. After 3 hours incubation at RT with rotation, the Dynabeads-Antibody-Antigen complex was placed on the magnet and the complex was washed 3 times with non-denaturing buffer. The complex was then resuspended in 100 µl of 2× sample buffer (125 mM Tris-HCl, 200 mM DTT, 20% glycerol, 6% SDS and 0.2% bromophenol blue) and incubated for 10 minutes at 70ºC. The tube was again placed on the magnet and the supernatant was collected. For western blotting, 50 µl of each total extract of the final eluate was used. The interaction between the NIS mutants and µ1A/B subunits was calculated as the fraction of protein in the immunoprecipitate *versus* the input. Data were normalized to the signal obtained in the immunoprecipitate with WT hNIS.

*Radio-iodide transport assay*

Polarized MDCK-hNIS and µ1B-KD-hNIS cells were assayed for iodide transport as described (13) with some modifications. After cell polarization on 12-mm Transwell filters, epithelial monolayer cells were incubated with 400 µl of Hank’s balanced salt solution containing 20 µM potassium iodide supplemented with carrier-free Na^125^I (specific activity 100 mCi/mmol I) in the upper or lower chambers. The radio-iodide-free chamber was incubated with 400 µl HBSS. Then, 10-µl aliquots were collected in each chamber at different time points up to 360 minutes. Every condition was performed in triplicate for each time point. Radioactivity was quantified in a ɣ-counter as counts per minute (cpm).

**References:**

**1.** Leoni SG, Sastre-Perona A, De la Vieja A, Santisteban P 2016 Selenium Increases Thyroid-Stimulating Hormone-Induced Sodium/Iodide Symporter Expression Through Thioredoxin/Apurinic/Apyrimidinic Endonuclease 1-Dependent Regulation of Paired Box 8 Binding Activity. Antioxid Redox Signal **24**:855-866.

**2.** Riedel C, Levy O, Carrasco N 2001 Post-transcriptional regulation of the sodium/iodide symporter by thyrotropin. J Biol Chem **276**:21458-21463.

**3.** Riesco-Eizaguirre G, De la Vieja A, Rodriguez I, Miranda S, Martin-Duque P, Vassaux G, Santisteban P 2011 Telomerase-driven expression of the sodium iodide symporter (NIS) for in vivo radioiodide treatment of cancer: a new broad-spectrum NIS-mediated antitumor approach. J Clin Endocrinol Metab **96**:E1435-1443.

**4.** Dohan O, De la Vieja A, Paroder V, Riedel C, Artani M, Reed M, Ginter CS, Carrasco N 2003 The sodium/iodide Symporter (NIS): characterization, regulation, and medical significance. Endocr Rev **24**:48-77.

**5.** Gravotta D, Deora A, Perret E, Oyanadel C, Soza A, Schreiner R, Gonzalez A, Rodriguez-Boulan E 2007 AP1B sorts basolateral proteins in recycling and biosynthetic routes of MDCK cells. Proc Natl Acad Sci U S A **104**:1564-1569.

**6.** Sastre-Perona A, Santisteban P 2014 Wnt-independent role of beta-catenin in thyroid cell proliferation and differentiation. Mol Endocrinol **28**:681-695.

**7.** Folsch H, Pypaert M, Schu P, Mellman I 2001 Distribution and function of AP-1 clathrin adaptor complexes in polarized epithelial cells. J Cell Biol **152**:595-606.

**8.** De La Vieja A, Ginter CS, Carrasco N 2004 The Q267E mutation in the sodium/iodide symporter (NIS) causes congenital iodide transport defect (ITD) by decreasing the NIS turnover number. J Cell Sci **117**:677-687.

**9.** Livak KJ, Schmittgen TD 2001 Analysis of relative gene expression data using real-time quantitative PCR and the 2(-Delta Delta C(T)) Method. Methods **25**:402-408.

**10.** Li Q, Lau A, Morris TJ, Guo L, Fordyce CB, Stanley EF 2004 A syntaxin 1, Galpha(o), and N-type calcium channel complex at a presynaptic nerve terminal: analysis by quantitative immunocolocalization. J Neurosci **24**:4070-4081.

**11.** Bolte S, Cordelieres FP 2006 A guided tour into subcellular colocalization analysis in light microscopy. J Microsc **224**:213-232.

**12.** Leoni SG, Kimura ET, Santisteban P, De la Vieja A 2011 Regulation of thyroid oxidative state by thioredoxin reductase has a crucial role in thyroid responses to iodide excess. Mol Endocrinol **25**:1924-1935.

**13.** De la Vieja A, Ginter CS, Carrasco N 2005 Molecular analysis of a congenital iodide transport defect: G543E impairs maturation and trafficking of the Na+/I- symporter. Mol Endocrinol **19**:2847-2858.
