# Supplementary material for: Basolateral Sorting of the Sodium/Iodide Symporter Is Mediated by Adaptor Protein 1 Clathrin Adaptor Complexes
Source: Thyroid. 2022 Oct 14;32(10):1259–70. doi: 10.1089/thy.2022.0163 (PMC9618391; doi:10.1089/thy.2022.0163)
Supplement: Supplemental data [file Supp_FigS1.docx]

**Supplementary Figure Legend**

**Figure S1. Cell morphology of MDCK-hNIS and µ1B-KD-hNIS cells.** Cells were plated on polycarbonate filters and analyzed for alterations in morphology by immunofluorescence. Localization of NIS (red), β-catenin (green) in the adherent junction, and ZO-1 (purple) in the tight junctions, and nuclei (blue) was revealed by staining with specific antibodies. Representative confocal immunofluorescence of xy sections and orthogonal xz plane views are shown. The region delimited by the white square with the dotted line in the xz sections has been enlarged in the bottom of each image. Scale bars, 10 µm.

**
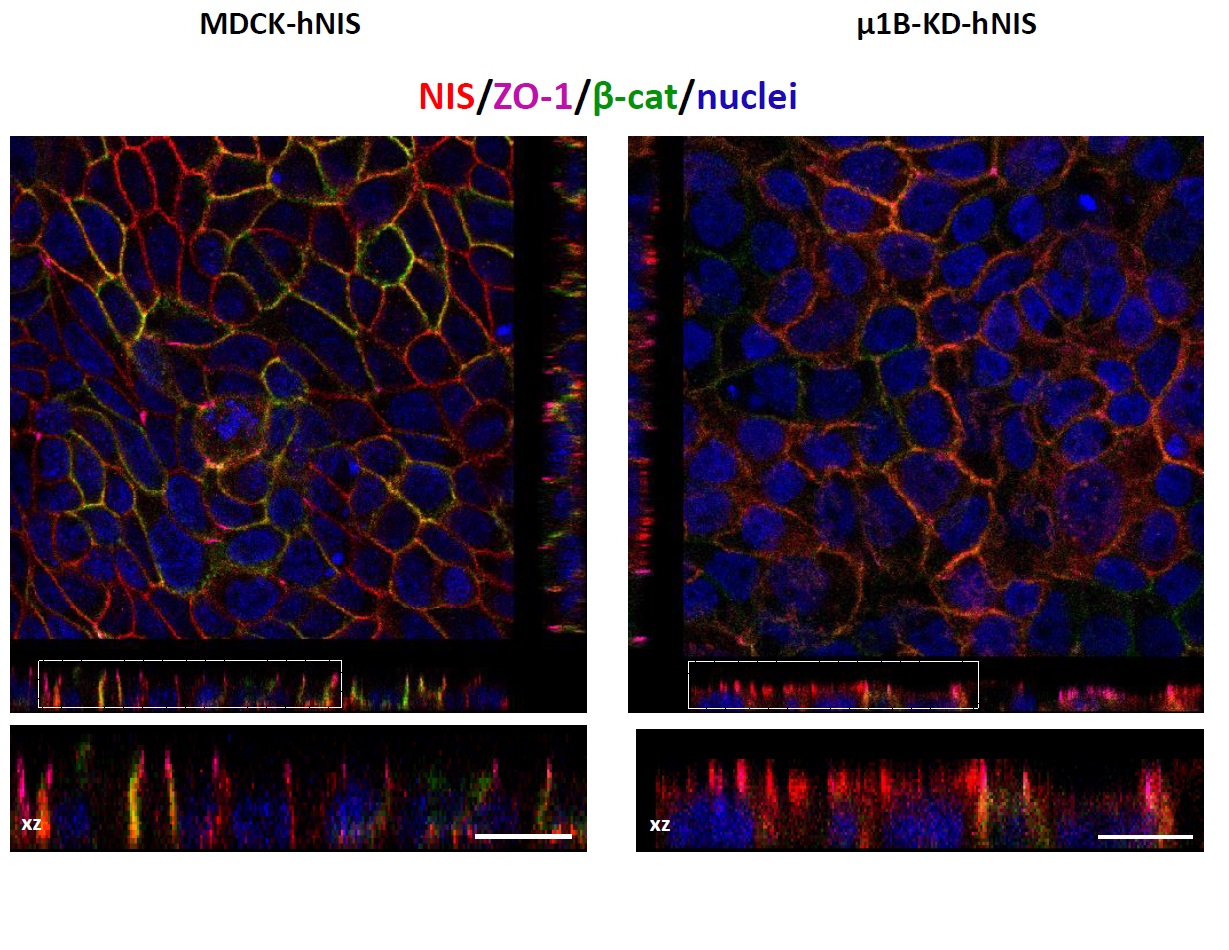
**
