# Supplementary material for: Basolateral Sorting of the Sodium/Iodide Symporter Is Mediated by Adaptor Protein 1 Clathrin Adaptor Complexes
Source: Thyroid. 2022 Oct 14;32(10):1259–70. doi: 10.1089/thy.2022.0163 (PMC9618391; doi:10.1089/thy.2022.0163)
Supplement: Supplemental data [file Supp_FigS2.docx]

**Figure S2. NIS localization in MDCK-hNIS and µ1AB-KD-hNIS by immunofluorescence microscopy.** MDCK-hNIS and µ1AB-KD-hNIS cells were plated on polycarbonate filters and analyzed for the subcellular distribution of NIS (red) and the apical marker gp135 (green). Representative confocal immunofluorescence of xy sections and orthogonal xz plane views are shown. The region delimited by the white square with the dotted line in the xz sections has been enlarged in the bottom to better observe the increase in intracellular NIS protein in µ1AB-KD-hNIS cells compared with control MDCK-hNIS cells. xy-z3 (basal region), xy-z15 (middle part of the cell) and xy-z25 (apical regions) section are shown. Intracellular NIS expression in the xy-15 is higher in µ1AB-KD-hNIS cells than in MDCK-hNIS cells. Scale bars, 20 µm.

**
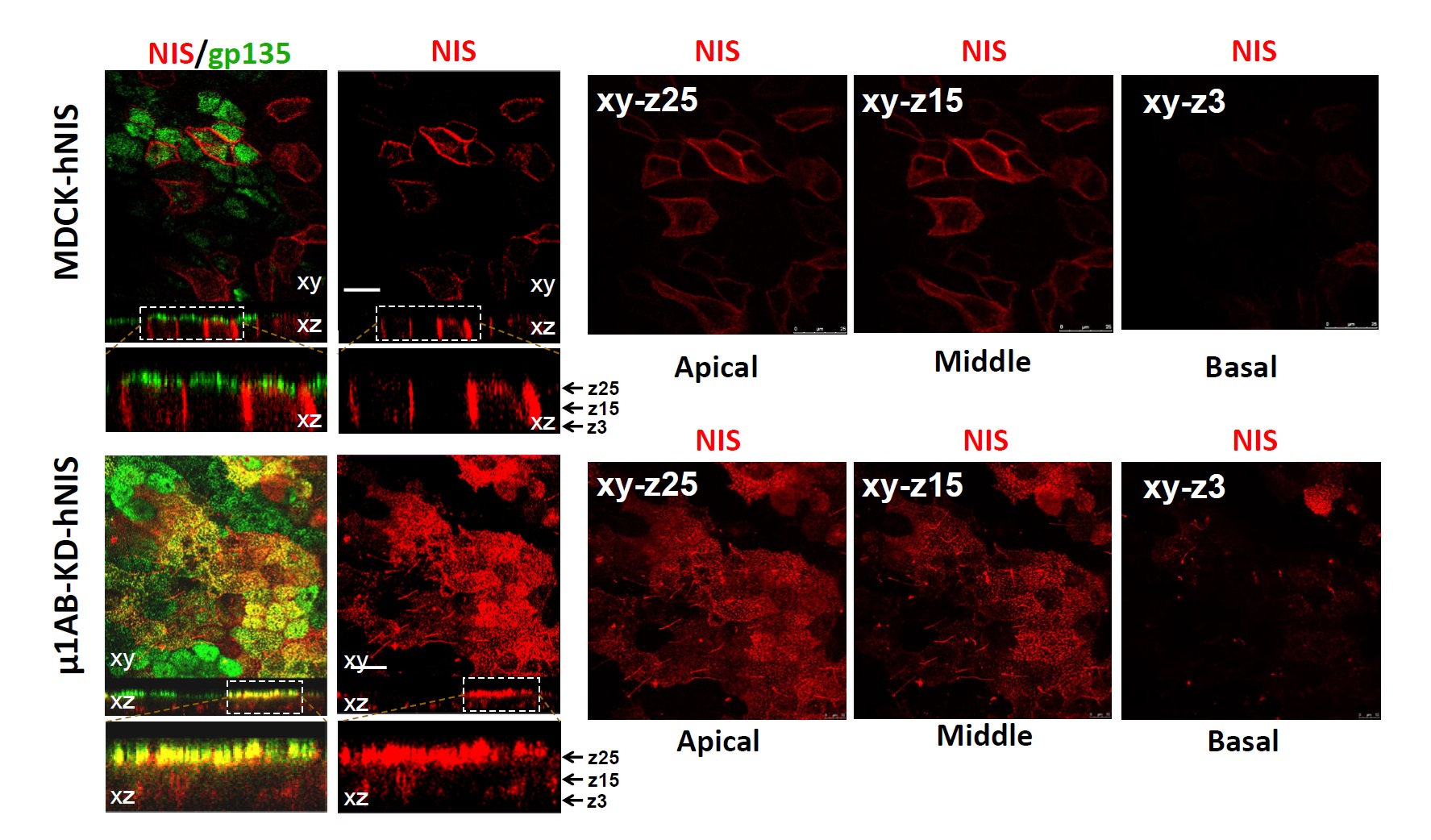
**
