# Supplementary material for: Basolateral Sorting of the Sodium/Iodide Symporter Is Mediated by Adaptor Protein 1 Clathrin Adaptor Complexes
Source: Thyroid. 2022 Oct 14;32(10):1259–70. doi: 10.1089/thy.2022.0163 (PMC9618391; doi:10.1089/thy.2022.0163)

**Figure S3. Analysis of *SLC5A5*, *AP1M1* and *AP1M2* expression and correlation in The Cancer Genome Atlas cohort.** A) Box plot of mRNA expression in papillary thyroid cancer (T, n=512) *versus* normal (N, n=337) thyroid tissue. B) Correlation of *AP1M1* and *AP1M2* with *SLC5A5* mRNA expression. The plots were generated using GEPIA software (http://gepia.cancer pku.cn) using data from The Cancer Genome Atlas data set of *Thyroid Cancer*.


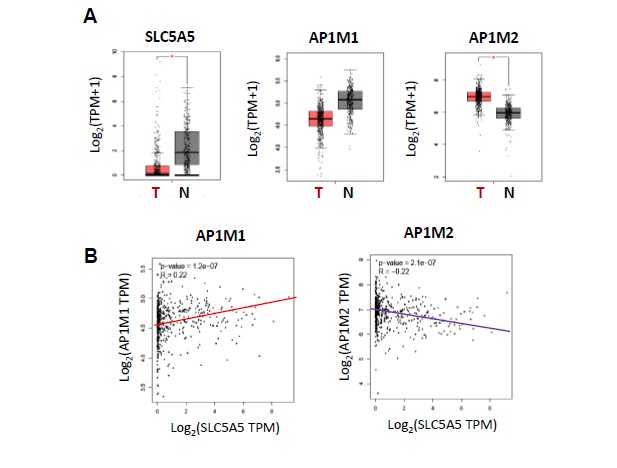

Supplement: Supplemental data [file Supp_FigS3.docx]
